# Risk factors and prevalence of antibodies for *Toxoplasma gondii* in diaphragmatic fluid in wolverines (*Gulo gulo*) from the Northwest Territories, Canada

**DOI:** 10.1016/j.fawpar.2019.e00056

**Published:** 2019-04-23

**Authors:** Rajnish Sharma, Sarah Parker, Brett Elkin, Robert Mulders, Marsha Branigan, Jodie Pongracz, Dale L. Godson, Nicholas C. Larter, Emily Jenkins

**Affiliations:** a Department of Veterinary Microbiology, Western College of Veterinary Medicine, University of Saskatchewan, 52 Campus Drive, Saskatoon S7N 5B4, Saskatchewan, Canada; b Centre for Applied Epidemiology, Large Animal Clinical Sciences, Western College of Veterinary Medicine, University of Saskatchewan, 52 Campus Drive, Saskatoon S7N 5B4, Saskatchewan, Canada; c Department of Environment and Natural Resources, Government of the Northwest Territories, 600, 5102-50th Avenue, Yellowknife X1A S8, NT, Canada; d Department of Environment and Natural Resources, Government of the Northwest Territories, P.O. Box 2749, Shell Lake, Inuvik X0E 0T0, NT, Canada; e Department of Environment and Natural Resources, Government of the Northwest Territories, PO Box 240, Fort Simpson, Northwest Territories, Canada

**Keywords:** *Toxoplasma gondii*, Seroprevalence, Wolverines, Northwest Territories, Canada

## Abstract

*Toxoplasma gondii*, a zoonotic food borne parasite that can infect almost all warm-blooded animals including people, and ranks 4th among 24 most significant global foodborne parasites listed by the World Health Organization/United Nations Food and Agriculture Organization (FAO/WHO, 2014). Exposure to *T. gondii* has been reported in wildlife and people in the Canadian North, despite low densities of feline definitive hosts. The ecology of this host-parasite system could be affected by changing climate and landscape in boreal and sub-Arctic regions, and surveillance data are critically needed. Wolverines are an economically and culturally important species in northern Canada due to their valuable fur. Fluid obtained from diaphragmatic muscle of 127 wolverines (*Gulo gulo*) were tested for antibodies to *T. gondii* using an enzyme linked immunosorbent assay (ELISA). A seroprevalence of 62% (Confidence Interval (CI): 53–71%) was observed. This result indicates high levels of exposure, likely either through environmental contamination with *T. gondii* oocysts shed by infected wild felids, or consumption of carcasses/offal of other intermediate hosts containing tissue cysts with bradyzoites in tissues. We examined factors associated with seropositivity, including age, sex, harvest location, harvest location with respect to treeline, and body condition index. Adult (≥2 years) wolverines had 5.2 times higher odds of being sero-positive than juvenile (<1 years) wolverines. The highest seroprevalence was observed in wolverines from Sahtu and South Slave regions. Proportion of sero-positive wolverines harvested above and below the tree line was not significantly different (60% vs 65%). Age was the only significant predictor of *T. gondii* exposure in wolverines (using logistic regression analysis); further studies should target larger sample sizes. This study is an example of how fluid from diaphragmatic muscle can be used for screening for *T. gondii* antibodies in wolverines. The diaphragm, commonly collected for screening for another food borne parasite, *Trichinella*, in wildlife harvested for human consumption, can be used for screening of *T. gondii* exposure in wildlife. Due to their predatory and scavenging lifestyle and high trophic level, wolverines could serve as a sentinel species for *T. gondii*.

## Highlights

• Using an ELISA, antibodies to *Toxoplasma gondii* were reported in wolverines (*Gulo gulo*) in the Canadian North.
• Significantly high seroprevalence was observed in adult wolverines than juveniles.
• ELISA can be carried out on fluid from diaphragmatic muscle for screening *T. gondii* antibodies in wolverines.
• Wolverines could serve as sentinel species for *Toxoplasma* exposure.

## Graphical abstract

*Toxoplasma gondii* exposure in wolverines from Northwest Territories.

Wolverine picture supplied by Emilie Bouchard (University of Saskatchewan).

**Figure:**
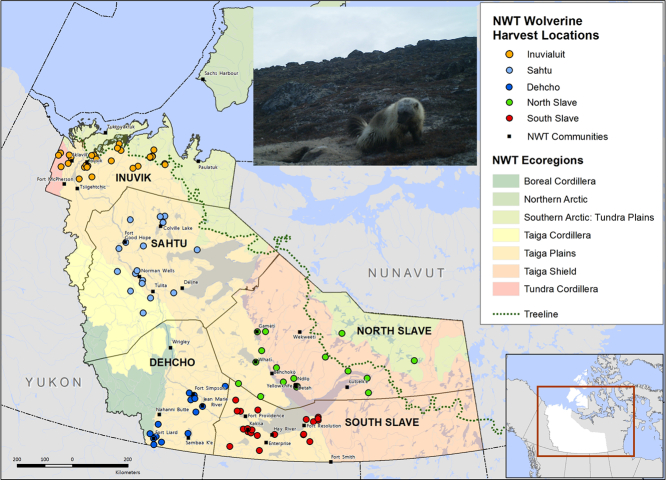

## 1 Introduction

Toxoplasmosis, an important food borne parasitic zoonosis, has been reported in almost all warm-blooded vertebrates, including livestock, wildlife, birds, marine mammals and humans (Dubey, 2010). *Toxoplasma gondii* ranks 4th among 24 most significant global foodborne parasites listed by the World Health Organization/United Nations Food and Agriculture Organization (FAO/WHO, 2014). *Toxoplasma gondii* has three infective stages in its life cycle: sporozoites (in oocyst), tachyzoites and bradyzoites. Felids are the only definitive host, where sexual reproduction of the parasite results in excretion of oocysts in feces. Intermediate hosts become infected by ingesting food or water contaminated with oocysts, which ultimately develop into bradyzoites in tissues. Ingestion of tissue cysts containing bradyzoites can also lead to *T. gondii* infection. Infection with *T. gondii* may occur by sexual (via semen) or transplacental routes. In humans, the infection is often asymptomatic, but clinical manifestation can range from mild to life-threatening, particularly among immunocompromised persons and fetuses (Dubey et al., 2005; Gajadhar et al., 2006; Halonen and Weiss, 2013; Hill and Dubey, 2002; Jones et al., 2009).

Exposure to *T. gondii* is common in people and animals in northern Canada, with seroprevalence as high as 65% in humans (Curtis et al., 1988), 62.5% in dogs (Salb et al., 2008) and 3–40% in wildlife [Caribou/Reindeer (*Rangifer tarandus*), Muskoxen (*Ovibos moschatus*) etc.] has been reported in some studies (Johnson et al., 2010; Kutz et al., 2001; Simon et al., 2011). Higher seroprevalence of *T. gondii* in muskox from the Northwest Territories (NWT), Canada than from Greenland (5% vs. 3%) (Clausen and Hjort, 1986; Kutz et al., 2000) as well as in reindeer from the NWT (40%) (Kutz et al., 2001) than Norway (1%) (Vikoren et al., 2004) and Alaska (6.6%) (Zarnke et al., 2000) has been reported. Wildlife can play an important role in the epidemiology of *T. gondii* infection, and has been documented or implicated as a source of the parasite in North America including northern Canada. An outbreak of congenital toxoplasmosis in northern Quebec, Canada was statistically linked to consumption of raw dried caribou meat and skinning of animals (McDonald et al., 1990). *Toxoplasma gondii* infection after consumption of raw venison was also documented in three US hunters (Sacks et al., 1983). An outbreak of toxoplasmosis occurred in British Columbia, Canada due to contamination of municipal drinking water with feces of cougars (*Felis concolor vancouverensis*) as well as domestic cats (Aramini et al., 1998).

Wolverines (*Gulo gulo)* are an economically important species in northern Canada because of their valuable fur. Wolverines are classified as vulnerable throughout their circumpolar range by the IUCN; however, the highest population densities in Canada occur in the mountains of the Yukon and Northwest Territories, where they are legally trapped (Naughton, 2012). Due to their high trophic level, wolverines could play the role of “bioaccumulators” of food borne parasites. Important characteristics of a sentinel animal host (Halliday et al., 2007; NRCCAMEH, 1991) may include measurable response (e.g. parasites in tissues, antibodies in blood), sufficient availability (population stability), and high levels of exposure. Their predatory and scavenging lifestyle and large home range predispose wolverines for exposure. Serological tests for the detection of antibodies to *T. gondii* are commonly used as screening tools for domestic and wildlife species. A variety of serological methods, including agglutination test (MAT), enzyme linked immunosorbent assay (ELISA) and indirect fluorescent antibody test (IFAT) have been used for detection of antibodies to *T. gondii* in wildlife species (Al-Adhami et al., 2016; Elmore et al., 2016). The MAT is the most widely used method, it is simple to use and does not require species-specific conjugates.

The first objective of this study was to determine the seroprevalence of *T. gondii* in wolverines via detecting anti-*T. gondii* antibodies in fluid collected from diaphragm tissues which are commonly collected in surveys for another foodborne parasite, *Trichinella* spp. The second objective was to assess the association between exposure to *T. gondii* and the risk factors such as age, sex, location and body condition index (BCI).

## 2 Materials and methods

### 2.1 Study area

Bordered by Nunavut to the east and the Yukon to the west, the NWT is the most populous of the three territories in Northern Canada, with an estimated population of 44,718 (Statistics-Canada, 2017). Climate varies from the southern part (subarctic climate) to the northern coast and the islands (polar climate). Communities depend on wildlife for traditional, cultural and economic values. Northwest Territories (NWT) harbors a variety of wildlife species including caribou (*Rangifer tarandus*), moose (*Alces alces*), muskox (*Ovibos moschatus*), mountain goats (*Oreamnos americanus*), Dall sheep (*Ovis dalli*), beaver (*Castor canadensis*), wolves (*Canis lupus*), foxes (*Vulpes vulpes*, *V. lagopus*), lynx (*Lynx Canadensis*), wolverines (*Gulo gulo*), and bears (*Ursus americanus*, *U. arctos*, *U. maritimus*).

### 2.2 Collection of samples

**Fig. 1:**
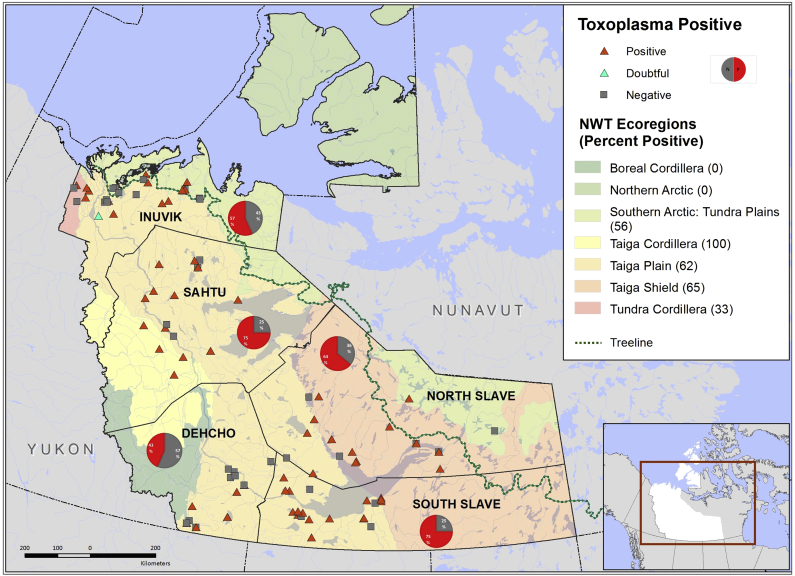
Map of the Northwest Territories showing the harvest locations of *Toxoplasama gondii* seropositive and seronegative wolverines in relation to region and ecoregion.

Wolverines were collected by trappers as part of the fur harvest during 2005–2012 in NWT (Fig. 1). Trappers submitted carcasses to Environment and Natural Resources, NWT for necropsy. Carcasses were collected from wolverines in four regions, Sahtu, Dehcho, North Slave, South Slave and Inuvik. These regions cover five different ecozones viz. Southern Arctic (14%), Taiga Cordillera (3%), Taiga Plains (65%), Taiga Shields (16%) and Tundra Cordillera (2%). Harvest location was further subdivided into above and below treeline. For wolverine harvested above treeline the straight-line distance between the harvest location and treeline was recorded. Sex and body condition index were determined at necropsy (Robitaille et al., 2012). Age was determined via cementum analysis of the canine tooth at a commercial laboratory (Matson's Laboratory LLC, Milltown, Montana, USA). Wolverines were classified as juveniles (<1 year), yearlings (1–2 years), and adults (≥2 year). Entire diaphragms were collected, frozen at −20 °C, and shipped to the Zoonotic Parasite Research Unit at the Western College of Veterinary Medicine, University of Saskatchewan.

### 2.3 Tissue fluid extraction

Diaphragms were placed individually in double plastic bags to avoid cross-contamination between samples and left at 4 °C for 24 h to thaw. Approximately, 1 ml of accumulated tissue fluid was removed from the bag and centrifuged at 1000 ×*g* for 5 min at 4 °C. The supernatant was stored at -20 °C until used for testing.

### 2.4 Enzyme linked immunosorbent assay (ELISA)

Tissue fluid samples were tested in duplicate for the presence of antibodies against *T. gondii* using a commercially available multi-species ID Screen Toxoplasmosis Indirect kit as per manufacturer's instructions (ID.vet, Grabels, France). Briefly, 100 μl of tissue fluid (at 1:2 in dilution buffer) were transferred to wells of an antigen coated ELISA plate and incubated for 45 min at room temperature. Wells were then washed 3 times and 100 μl 1× multispecies peroxidase (HRP) conjugate was added. The ELISA plate was incubated for 30 min at room temperature and then washed three times. Substrate solution (100 μl) was added to each well and the plate was incubated in the dark for 15 min at room temperature. After addition of 100 μl stop solution, optical density (O.D) values at 450 nm were recorded using an ELISA automated plate reader (Spectramax, Molecular Devices). Tests were considered valid if the mean value of the positive control OD was >0.350 and if the ratio of the mean OD values of the positive and negative controls was >3.

S/P percentage was calculated using controls provided by the manufacturer as per the following formula.

$$S/P\%=\;\left({\mathrm{OD}}_{\text{sampl}}-{\mathrm{OD}}_{\text{negative control}}/{\mathrm{OD}}_{\text{positive control}}-{\mathrm{OD}}_{\text{negative control}}\right)\times 100$$

Samples presenting an S/P ratio ≤ 40% were considered negative. If the S/P ratio was between 40% and 50%, test outcome was considered doubtful and the sample was omitted from statistical analysis. Samples with an S/P ratio ≥ 50% were considered positive.

In addition to positive and negative controls provided in the kit, we included diaphragmatic fluid from four and two wolverines detected positive and negative for *T. gondii* DNA in heart tissue using magnetic capture PCR (Bachand et al., 2018; Sharma et al., 2018); these tissue-positive and -negative wolverines showed seropositivity and seronegativity, respectively on commercial available ELISA kit.

### 2.5 Statistical analysis

Seroprevalence was calculated from the proportion of sero-positive results of those tested and is presented with 95% confidence intervals. One sample had a doubtful result and was not included in statistical analysis. Possible associations between outcome variable (either positive/negative for antibodies against *T. gondii*) were evaluated by binary logistic regression. The following predictors were evaluated for inclusion in a final multivariable model using univariable binary logistic regression analysis: Age (juvenile, yearling and adult), sex (female and male), harvest location (Dehcho, Sahtu, Inuvik, North Slave, and South Slave regions), harvest location with respect to tree line (above or below tree line) and body condition (continuous factor). A relaxed level of significance (*p* ≤ 0.20) was used to identify variables. Stepwise forward multivariable regression analysis was performed to include risk factors and confounders in final model. Goodness of fit of the final model was evaluated by Hosmer Lemeshow test. Variables associated at a significance level of *p* < 0.05 were retained in the model. To estimate the degree of the association between each predictor and *T. gondii* sero-status, odds ratios and respective 95% confidence intervals were calculated. All statistical analyses were performed using IBM SPSS (ver. 24; Armonk, New York, USA).

### 2.6 Ethical approval

As animals were harvested for purposes other than research, this was considered Category A and exempt from Animal Research ethics review at the University of Saskatchewan. We worked closely with the Government of the Northwest Territories for wildlife research and export permits.

## 3 Results

### 3.1 Descriptive analysis and anti *T. gondii*–antibody prevalence

A total of 127 wolverines covering a range of sex and age were trapped for this study, 112 (88%) from below tree line and 15 from above treeline. The mean age and BCI of sampled wolverines was 2.07 years (SD 2.49 range, 0–11) and 10.82 (SD 6.6, range, 1–40), respectively; male to female ratio was 3:2 (male: female). Antibodies to *T. gondii* were found in 62.2% (79/127; 95% CI 53.17–70.65) of the analyzed wolverines. Antibodies to *T. gondii* were detected most frequently in adult wolverines (81%, 42/52) followed by yearlings (59%, 17/29) and juvenile wolverines (44%, 20/45). Nearly equal proportion of females (66%, 33/50) and males (61%, 46/76) were -anti *T. gondii* -antibody–positive. Sero-positive wolverines were found in all five regions sampled, with the highest seroprevalence from Sahtu and South Slave regions (both 75%, 18/24) followed by North Slave (64%, 16/25), Inuvik (57%, 17/30) and Dehcho region (43%, 10/23) Fig. 1. Almost equal proportions of wolverines from above (60%, 9/15) and below (63%, 70/111) the tree lines were positive. Mean straight line distance was higher (26 km) in sero-negative than seropositive wolverines (19 km). Among ecozones, highest seroprevalence was reported in wolverines from Taiga Cordillera (4/4, 100%) followed by Taiga shield (13/20, 65%), Taiga Plains (51/81, 62%), Southern Arctic (10/18, 56%) and Tundra Cordillera (1/3, 33%). Year of collection and ecozones were not included in logistic regression analysis due to uneven sampling during different years of collection, and association between harvest location and ecozones,

### 3.2 Association with the risk factors using regression models

On univariable logistic regression, age (*p* = 0.02) and location (*p* = 0.139) appeared to be associated with exposure to *T. gondii* (Table 1). After multivariable regression, only age was significantly associated with sero-positivity. The odds of presence of anti-*T. gondii* antibodies were five times (odds ratio = 5.2, 95% CI (2.12–13), *p* = 0.002) higher in adult wolverines than in juvenile wolverines.

**Table 1:** Univariable analysis on variables associated with *Toxoplasma gondii* sero-positivity among wolverines, *Gulo gulo* of the Northwest Territories.

Variable	P (%)	n	OR	95% CI	*P* value
**Age**					0.02
Juvenile (ref)	20 (44)	45			
Yearling	17 (59)	29	1.77	0.689–4.55	0.236
Adult	42 (81)	52	5.25	2.12–12.99	0.0
**Sex**					
Female (ref)	33 (66)	50			
Male	46 (61)	76	0.79	0.375–1.663	0.535
**Harvest location**					0.139
Dehcho (ref)	10 (43)	23			
Inuvik	17 (57)	30	1.7	0.568–5.086	0.343
North Slave	16 (64)	25	2.31	0.724–7.375	0.157
Sahtu	18 (75)	24	3.9	1.131–13.454	0.031
South Slave	18 (75)	24	3.9	1.131–13.454	0.031
**Location with respect to tree line**					
Below tree line (ref)	70 (63)	111			
Above tree line	9 (60)	15	0.879	0.292–2.646	0.818
**BCI**		126	0.96	0.916–1.025	0.272

n = number of wolverines tested, P (%) = number of positive animals (% age positive), OR = Odd ratio.

## 4 Discussion

We successfully detected antibodies against *T. gondii* in diaphragmatic fluid from wolverines, demonstrating the utility of this approach in wildlife (especially in remote regions) from which sera are not routinely available. We reported high seroprevalence of *T. gondii* in wolverines from the NWT in comparison to seroprevalence reported in other wildlife species in NWT [5% in Muskoxen, 3%–40% in Caribou/Reindeer, 22% in Harbour Seal (*Phoca vitulina*), 10% in Ringed Seal (*Phoca hispida*), and 10% in Bearded Seal (*Erignathus barbatus*)] (Johnson et al., 2010; Kutz et al., 2000; Kutz et al., 2001; Simon et al., 2011), although results were on par with those observed in dogs in a community in the southern NWT (Salb et al., 2008). The 62% of the sero-positive wolverines from NWT, which is higher than the seroprevalence reported in two previous reports of *T. gondii* exposure in wolverines in Canada: 42% of 41 wolverines in Nunavut (Reichard et al., 2008) and none of 20 in BC (Philippa et al., 2004). Variation in seroprevalence among wolverines from these three different provinces/territories could be due to differences in serological methods [ELISA vs MAT in, current study and (Philippa et al., 2004), MAT (Reichard et al., 2008)], specimens used [serum in (Philippa et al., 2004), spleen exudates in (Reichard et al., 2008)], diaphragmatic fluid in current study and dietary habits of wolverines from different locations. We chose an ELISA, which requires less time, and results are more objective in comparison to MAT. As species-specific conjugates are not available for wolverine, we used a commercially available ELISA kit (ID.vet, Grabels, France) with A/G conjugates, which has been previously used in other wildlife species (Racka et al., 2015; Roqueplo et al., 2011). This ELISA had sensitivity and specificity equal or superior to two serological tests (MAT, and IFAT) using magnetic capture qPCR as a reference test. We used a 1:2 dilution as a cut off as recommended by the manufacturer and confirmed in wolverines using different dilutions of meat juice (Sharma et al., 2018). As sensitivity and specificity of ELISA using diaphragmatic fluid to detect anti-*T. gondii* antibodies is unknown, we reported apparent seroprevalence.

There are many reports of prevalence of antibodies to *T. gondii* in blood collected from immobilized or live-trapped wildlife by biologists and researchers (Elmore et al., 2016; Johnson et al., 2010); however, sera are rarely available from trapped and hunted wildlife, which are most important from the perspective of human exposure. We adapted this test for use on tissue fluid as sera were not available, and previous studies reported a high degree of agreement between MAT and ELISA tests performed on sera and tissue fluid (Gamble et al., 2005; Halos et al., 2010). As tissue fluid has fewer antibodies than serum, we used a lower dilution (1:2) as compared to serum (1:10) (Nockler et al., 2005; Racka et al., 2015). This study suggests that diaphragmatic fluid is a suitable substrate for tests for antibody to *T. gondii*. Diaphragm is frequently collected in wildlife being screened for another food-borne parasite, *Trichinella* spp. Therefore, diaphragm and its fluid can be a convenient sample for testing *Trichinella* spp. infection, and *T. gondii* exposure, respectively. Our in-house controls (tissue fluid obtained from diaphragms of wolverines of known *T. gondii* status) performed well (tissue-positives and -negatives were sero-positives and -negatives, respectively) with a commercial ELISA kit, and the use of wildlife species-specific controls whenever possible is recommended. Further studies are required on validation of serological tests for detection of *T. gondii* antibodies in tissue fluid/sera of wolverines.

Our finding that significantly higher proportion of seropositive adult than juvenile wolverines is not surprising due to the longevity of antibodies (potentially lifelong) and more cumulative exposure to food or water contaminated with oocysts and/or consumption of meat having tissue cysts. However, the dynamics of anti-*T. gondii* antibodies are not known in wolverines. Higher prevalence in adult wolverines is in agreement with surveys conducted in other wildlife species from different geographical areas, e.g. wolves (*Canis lupus*) from Alaska, (Zarnke et al., 2000); maned wolves (*Chrysocyon brachyurus*) from Brazil, (Vitaliano et al., 2004); Eurasian otter (*Lutra lutra*) from England and Wales, (Chadwick et al., 2013)]. On the other hand, our findings are not consistent with those reported in wolverines from Nunavut (Reichard et al., 2008), in which apparently smaller percentage of adult wolverines (37%, 11/30) were positive for antibodies to *T. gondii* than juveniles (55%, 6/11). No significant association between age and *T. gondii* exposure in wolverines from Nunavut was reported (Reichard et al., 2008), nor in feral cats from Prince Edward Island, Canada (Stojanovic and Foley, 2011). A slight difference of seroprevalence among males and females was not significant (despite a male bias in harvest), similar to that reported in other wildlife surveys (feral cats, (Stojanovic and Foley, 2011), e.g. Eurasian otter, (Chadwick et al., 2013); grizzly and black bears, (Chomel et al., 1995); caribou, (Kutz et al., 2001). The body condition of wolverines in our study did not differ significantly with exposure to *T. gondii*, similar to findings in free roaming cats and dogs from Mexico (Ortega-Pacheco et al., 2017) as well as in wild and domestic cats from France (Afonso et al., 2013). Exposure to *T. gondii* in wolverines across all sites (region-specific seroprevalence ranged from 44 to 75%) indicates widespread transmission of the parasite across NWT, and geographical differences were not significant, likely as a result of small sample size.

Lynx (*Lynx canadensis*) are the only wild felid present in northern Canada and they are rarely seen above the treeline; thus, we hypothesized decreased opportunity for exposure to oocysts above the treeline (i.e. some parts of Inuvik and North Slave). Among 15 wolverines harvested above the tree line (13 from Inuvik and 2 from North Slave), mean straight-line distance from tree line was 22 km (range 4–99 km). Both the wolverines [IDs: W2010–086 (71 km) and NS06–92 (99 km)] from North Slave had highest straight line distance and were positive and negative for anti*-T. gondii* antibodies, respectively. Wolverine (ID: TU11/02) from Inuvik harvested at highest straightline distance was 33 km above the tree line and was sero-positive. Presence of sero-positive wolverines above the tree line indicates either environmental contamination with oocysts or presence of potential intermediate hosts (caribou and muskoxen). Also, as home range of wolverines varies from 100 to 800 km^2^, possibility of migration of sero-positive wolverines cannot be ignored. An association between the presence of antibodies to *T. gondii* and proximity to the treeline has been reported in wolverines from Nunavut (Reichard et al., 2008) and in caribou and muskoxen (Kutz et al., 2000; Kutz et al., 2001). Future studies could evaluate the spatio-temporal occurrence of *T. gondii* infection in wild carnivores and potential intermediate hosts in NWT relative to treeline, explore new serological methods that can distinguish exposure to oocysts vs tissue cysts, and compare genotypes of *T. gondii* circulating in wolverines and Canadian lynx. Further studies are needed on *T. gondii* in domestic and wild felids in NWT as well as their relative contribution to environmental oocyst contamination.

Finally, little is known about the effects of *T. gondii* in free ranging wildlife. Clinical toxoplasmosis has been reported in wild carnivores e.g. foxes (Dubey et al., 1990; Dubey and Pas, 2008), wild boars (Calero-Bernal et al., 2013), and raccoons, *Procyon lotor* (Dubey et al., 1992). An outbreak reported in mink (close relative of wolverines) resulted in abortions and neonatal mortalites occurring in 26% of the female mink (*n* = 7800) (Frank, 2001). Fatal toxoplasmosis has been reported in sea otters (*Enhydra lutris*), brown hare (*Lepus europaeus* P.), Eurasian red squirrels (*Sciurus vulgaris*), Pallas' cats (*Otocolobus manul*) (Kreuder et al., 2003, Brown et al., 2005, Gustafsson and Uggla, 1994, Jokelainen and Nylund, 2012). Further studies are required to see if *T. gondii* is responsible for pathology and decreased reproductive success in wolverines.

Detection of antibodies to *T. gondii* in animals indicates prior exposed to the parasites but does not always indicate if it is infected (Kornacka et al., 2016). Therefore, our study can only speak to exposure, and bioassays (cat and mouse) or molecular methods are needed to determine tissue status and therefore any hazard to trappers/hunters handling fresh tissues of wolverines. However, as association between skinning and human exposure to *T. gondii* has been reported (McDonald et al., 1990), hunters/trappers should avoid unintentional ingestion of tissues or fluids and follow good sanitary practices while processing wolverine carcasses (Hill et al., 2014). It is not recommended to leave carcasses and offal where they can be scavenged, perpetuating the life cycle of the parasite, especially near human habitations. In addition to wolverines, other wild animals, for example, moose (*Alces alces*) (Remes et al., 2018) and wild boar (*Sus scrofa*) (Ranucci et al., 2013) could act as sentinels for the exposure to *T. gondii*, however, moose, which are browsing herbivores, serve as indicators of oocyst transmission, while more omnivorous species such as boar and wolverine may also serve as indicators of overall transmission, including consumption of tissue cysts via carnivory.

## 5 Conclusion

Our results indicate that *T. gondii* circulates in wolverines throughout NWT. Due to their apex position in the food chain, dietary habits, and reported high seroprevalence, wolverines can serve as useful sentinels of risks of transmission to other wildlife and people from their shared environment in the Canadian north. We report the successful application of a commercial serological test to a sample substrate (diaphragmatic fluid) commonly available in harvested wildlife, which may have food safety applications. Finally, we found, as expected, that older animals were more likely to be exposed to *T. gondii*, while other risk factors for seropositivity should be explored with a larger sample size.
